# Filling the gap: artificial intelligence-driven one health integration to strengthen pandemic preparedness in resource-limited settings

**DOI:** 10.3389/fpubh.2025.1707306

**Published:** 2025-12-10

**Authors:** Disha Mukherjee, Ketul Sagar, Rea Maja Kobialka, Prakash Ghosh, Manfred Weidmann, Behrouz Alizadeh Savareh, Siddhartha Narayan Joardar, Uwe Truyen, Ahmed Abd El Wahed, Arianna Ceruti

**Affiliations:** 1 Indian Institute of Technology Kharagpur, Kharagpur, India; 2 Institute of Animal Hygiene and Veterinary Public Health, Leipzig University, Leipzig, Germany; 3 Department of Veterinary Microbiology, West Bengal University of Animal and Fishery Sciences, Kolkata, India

**Keywords:** One Health, artificial intelligence, pandemic preparedness, infectious diseases, resource-limited settings

## Abstract

Emerging zoonotic pathogens like SARS-CoV-2 and Nipah virus demonstrate the critical need for integrated surveillance systems connecting human, animal, and environmental health. This review examines how artificial intelligence can address One Health integration gaps in pandemic surveillance, focusing on resource-limited settings. While global digitization levels now support Artificial Intelligence (AI)-powered platforms, LMICs face barriers including limited resources and fragmented data systems. Current AI tools remain domain-specific and designed for high-income settings, limiting its applicability to pandemic preparedness in low-resource settings. Existing AI-tools and gaps are described and put into perspective within an AI-driven One Health framework, specifically for LMICs. The framework exemplifies resource optimization, governance, sectoral collaboration, capacity building, health system integration, geographic accessibility, and prioritization. The framework also features an exemplified dual solution combining Graph Neural Networks for integrated risk assessment with offline-first mobile applications for community surveillance. AI technologies offer substantial potential for pandemic preparedness through automated data harmonization, predictive modeling, and resource optimization. However, successful implementation requires concurrent digitization, cultural adaptation, and local capacity building. Prioritizing mobile solutions with minimal infrastructure requirements alongside community engagement will be essential for creating equitable AI-based surveillance systems in LMICs.

## Graphical abstract

**Figure:**
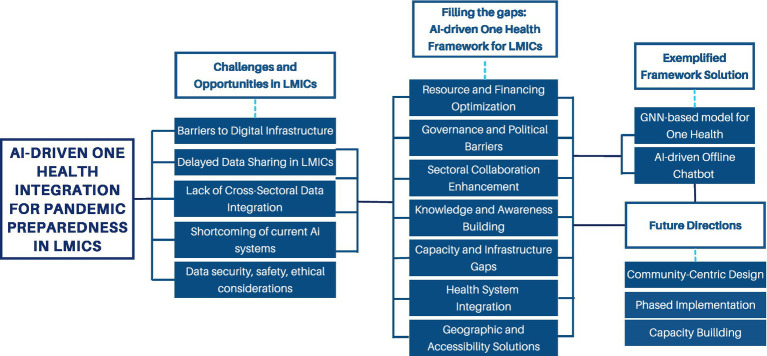

## 1 Introduction

Emerging zoonotic diseases such as COVID-19, Nipah virus disease, and avian influenza are clear reminders that outbreaks often begin at the intersection of human activity, animal health, and environmental change. One Health is an integrated, collaborative approach that recognizes the interconnection between human health, animal health, and environmental health. The concept promotes coordination across multiple sectors and disciplines to achieve better health outcomes for all (1). Around 60% of known infectious diseases and up to 75% of new or emerging infectious diseases have zoonotic origin (2). It emphasizes that the health of people, animals, and ecosystems are closely linked and interdependent. The concept, however, extends far beyond zoonoses to encompass numerous interconnected health challenges: antimicrobial resistance, food safety and security, social determinants, and environmental health (3).

The One Health integration is outlined as a key component of a pandemic preparedness framework. However, most surveillance systems across the world—including those recommended in the WHO’s *Future Surveillance* report (4)—fail to integrate the One Health domains effectively (5, 6). Key implementation principles for pandemic preparedness include adopting a multi-hazard approach, relying on evidence-based practices, prioritizing equity, and committing to continuous improvement (5).

Human health data, veterinary records, and environmental indicators are collected in silos, resulting in missed opportunities for early detection of spillover risks. While high-income countries have started using artificial intelligence (AI)-powered systems for genomic surveillance and outbreak prediction (7–9), these tools remain focused on specific domains and rarely achieve the integrated One Health perspective required for comprehensive pandemic preparedness. In low and middle-income countries (LMICs), this problem is further exacerbated by digital infrastructure gaps, fragmented data collection methods, and limited connectivity (4). However, both digital and AI tools have been reported. Digitalization efforts have been described, such as mobile phone–based survey (10, 11) and web-based and/ or native-app digital health tools across the large-scale vaccination process (12). A promising AI-tool is federated learning, which is a distributed machine learning paradigm that enables multiple participants to collaboratively train a shared model without centralizing their raw data. It has been used for chest imagining without the need of raw patient data sharing (13). Although they offer promise, they too suffer from lack of interoperability and limited reach to rural and animal health sectors.

There is a pressing need for scalable, affordable AI solutions that can bridge these divides. This paper reviews current needs, gaps and a framework for One Health-oriented and AI-driven pandemic preparedness for LMICs.

## 2 From digitization to artificial intelligence

One Health integration that links human, animal, and environmental health requires intelligent systems capable of identifying patterns across these interconnected domains. Traditional surveillance approaches, which treat each sector independently, are insufficient for detecting emerging zoonotic threats in time. Both digital and AI-driven tools have been described for diverse applications within these sectors. However, digitalization and AI tools serve different purposes in modernizing processes. Digitalization is the broader transformation of converting analog or manual processes into digital formats. This includes moving from paper records to digital databases, implementing electronic workflows, digitizing documents, or adopting basic software systems (14). The focus is on efficiency, accessibility, and standardization of existing processes without necessarily making them “smart.” AI tools add intelligence and automation to digital processes. They can analyze patterns, make predictions, automate decision-making, join heterogeneous data, and learn from it, providing capabilities like natural language processing, image recognition, predictive analytics, and autonomous task execution (15–18).

Current global digitization levels provide sufficient technological infrastructure to support the development of AI tools for One Health integration. Digital health infrastructure has expanded rapidly, with over 5.16 billion internet users worldwide as of 2023, representing 64.4% of the global population, and healthcare digitization accelerating significantly post-COVID-19 (19). Additionally, the proliferation of digital health technologies, including electronic health records, mobile health applications, and Internet of Things (IoT)-enabled monitoring systems across human, animal, and environmental health sectors, creates the necessary data ecosystem for potential AI-powered One Health platforms (20, 21). An example of powerful AI-tools that can improve intelligence of surveillance systems are Graph Neural Networks (GNNs), which offer the ability to learn complex relationships and make predictions from diverse, multi-source datasets (22). GNNs are deep learning models designed to operate on graph-structured data, where information is represented as nodes connected by edges. Unlike traditional neural networks that work with grid-like data (images, sequences), GNNs can handle irregular, non-Euclidean structures like social networks, molecular compounds, or knowledge graphs (23). They have been applied to COVID-19 spread forecasting (24–26). It is imaginable that GNNs could, therefore, integrate diverse datasets such as animal migration trends, land use changes, and localized human symptoms to forecast spillover risks and create integrated early warning systems.

In LMICs the application of AI is hampered not by its capabilities, but by the lack of digital health infrastructure (27). Much of the critical data—especially from rural clinics, veterinary outposts, and environmental field stations—remains on paper or in incompatible formats, making it inaccessible to AI systems. While high-income countries have started to benefit from integrated digital surveillance platforms, LMICs often face delayed outbreak response due to fragmented and analog data systems. Despite this, promising but single AI examples have emerged, from mobile chatbots using natural language processing (NLP) that can help triage symptoms in rural areas, to convolutional neural networks-vision transformers architecture for enhanced malaria detection (28). In an additional development stream, federated learning to enable privacy-conscious AI development in African health systems has been trialed (10, 12, 13, 28). These examples show that AI tools can work in LMICs—but without concurrent efforts to digitize data across health sectors, their impact to address pandemic preparedness remains limited. Therefore, digitization must be prioritized alongside AI tool development to enable scalable disease surveillance in LMICs.

In digitally mature environments, the implementation of One Health relies on collaboration among a diverse range of cross-sectoral stakeholders (29, 30). Within the human health sector, public health authorities, healthcare providers, and epidemiologists work together to collect and interpret health data that can inform real-time prediction and intervention (31). The animal health sector plays an equally important role, with veterinary services, livestock health agencies, and wildlife surveillance networks monitoring animal diseases that may pose risks to humans. The environmental sector contributes by providing data from environmental monitoring agencies, biodiversity researchers, and remote sensing organizations that track ecological and climatic factors influencing disease dynamics (32).

These sectors are supported by digital and technological stakeholders—such as AI developers, data scientists, and ICT infrastructure providers—who build and sustain the digital systems and analytical tools that facilitate integration and real-time analysis throughout the One Health framework. These efforts are supported by governance and policy stakeholders, such as national governments, international organizations (including WHO, FAO, WOAH, and UNEP), and local NGOs, who ensure coordination, resource allocation, and policy alignment (33). Academic and research institutions further contribute by advancing transdisciplinary research in the field.

Together, these stakeholders form an interconnected ecosystem that bridges human, animal, and environmental health with digital innovation and governance (33). In low- and middle-income countries (LMICs), this collaboration is especially crucial, as digitization and AI tools must be co-developed with local actors to ensure inclusivity (34).

## 3 Challenges and opportunities in LMICs

The use of AI can have significant advantages for One Health integration into pandemic preparedness. However, pivotal gaps are yet to be filled (Table 1).

**Table 1:** The key advantages and challenges of AI Tools for pandemic preparedness.

Advantages	Challenges
Early outbreak detection via real-time data processing	Limited data integration across human, animal, and environmental domains
Predictive modeling for hotspot identification and resource allocation	Data silos and lack of interoperability between sectors
Automated surveillance using Natural Language Processing, thermal imaging, and social media scans	Reliance on structured clinical data, excluding informal or analog sources
Faster diagnostics through image-based and genomic AI tools	Low model generalizability to LMIC settings due to biased training data
Scalable deployment via mobile apps and cloud platforms	Connectivity and infrastructure gaps, especially in LMICs
Federated learning enables local model training without data sharing	Lack of technical expertise and local AI development capacity
Enhanced decision support with real-time dashboards and alerts	Neglect of One Health factors like wildlife and environmental signals
Rapid vaccination planning via digital ID and scheduling systems	Privacy and data governance concerns in cross-border health surveillance

### 3.1 Barriers to digital health infrastructure in LMICs

LMICs face severe infrastructure challenges that prevent the deployment of AI-powered surveillance. There are current efforts to introduce digital surveillance tools. Challenges include for example the lack of interoperability in the sentinel syndromic surveillance system in preparedness and response programs (35). Even if there are tools in use, most of them do not offer a comprehensive set of attributes, resulting in the need for public health workers having to use multiple tools in parallel (36).

Limited financial resources represent the most fundamental barrier, as many LMICs struggle to allocate sufficient funding for basic healthcare services, let alone sophisticated digital infrastructure investments that require substantial upfront capital and ongoing maintenance costs (37, 38).

WHO’s report highlights unreliable electricity, patchy internet coverage, and the continued use of paper records as barriers to timely outbreak reporting (4). This undermines the reliability of digital systems and limits the deployment of IoT sensors, mobile devices, and cloud-based platforms essential for integrated health monitoring. Limited technical expertise and data governance and protection frameworks have also been pointed out as key obstacles, for example for federated AI model training in African hospitals (13, 39, 40). Interoperability issues further complicate digital health implementation, as legacy systems from different donors and development partners often use incompatible standards and protocols, creating fragmented digital ecosystems that impede data integration and analysis (41).

### 3.2 The cost of delayed data sharing in LMICs

Communities living in isolated rural communities face regular exposure to diseases that spread from animals to humans through their close contact with livestock and wildlife, yet they receive significantly less support and have limited access to healthcare services (42). A digital and mobile link to these areas would enhance the public health response.

Thus, the digital lag has direct consequences for public health. In Bangladesh, Nipah virus spread undetected because livestock morbidity data was logged manually, while data from remote settings was often not available—a failure WHO cites as common in LMIC settings (43). Most AI tools are tested in high-income regions, leaving LMICs without validated, scalable solutions for outbreak prediction and response (9).

### 3.3 Integrating cross-sectoral data for AI in pandemic preparedness

Recent pandemic events demonstrate the critical One Health interconnection, yet WHO notes that failing to integrate these data streams leaves surveillance systems blind to early warnings (4). The Nipah outbreaks in Bangladesh exemplify this challenge, where ecological changes and bat behavior remained unlinked to human health alerts due to data silos, causing delayed responses (44). Even in high-resource settings, these domains remain largely separated despite technological advances. A major obstacle is dataset incompatibility across sectors: clinical records use structured electronic formats, veterinary reports rely on inconsistent systems, and environmental data requires specialized geospatial processing (45). WHO warns that agencies responsible for different health domains rarely share interoperable platforms (4). While AI has been applied to outbreak detection, comprehensive One Health models that simultaneously process animal migration, livestock health, and human disease data remain undeveloped, particularly in LMICs (29). High-income countries have successfully deployed AI within human health systems—using machine learning for hospital data streams, genomic sequencing with mobility data, and tools for outbreak forecasting and social media monitoring. Current models predominantly focus on human data pipelines, with environmental variables often coarse rather than specific wildlife interaction data. Hence, spill-over risk calculation models should be continuously improved (46). Until these gaps are addressed, AI’s pandemic preparedness potential remains underutilized (47).

### 3.4 Shortcomings of current LMIC AI systems

Despite isolated successes, AI frameworks in LMICs remain fragmented and heavily reliant on human health data, overlooking crucial veterinary and environmental signals (48, 49). Moreover, the absence of localized AI development capacity forces LMICs to depend on solutions designed for high-income settings, which may not align with local realities or infrastructural constraints (27, 50). For example, AI models or mobile apps developed in urban centers may perform poorly in remote areas with different disease ecologies and health behaviors (51). This technology gap persists because of limited investment in regional AI expertise-building and a lack of scalable, open-source platforms adaptable to LMIC-specific needs. As a result, most AI tools deployed in LMICs remain externally driven, risking low adoption or relevance in local public health ecosystems (52).

### 3.5 Data security, safety, and ethical considerations for AI applications

Limited cybersecurity infrastructure, outdated systems, and scarce technical expertise make AI tools for pandemic preparedness highly vulnerable to breaches (51, 53, 54). Different disease presentations and pathogen strains in LMIC contexts can significantly affect AI accuracy, creating risks of algorithmic bias leading to misdiagnosis or inappropriate treatment recommendations (55, 56). System failures may leave healthcare workers without fallbacks when infrastructure faces constraints such as inadequate funding, outdated equipment, and shortages of technical expertise.

AI in healthcare raises ethical challenges around equity, consent, exploitation, and cultural sensitivity in LMICs. Without deliberate safeguards, AI may widen health disparities, benefiting urban or wealthy populations while excluding those with language barriers, low digital literacy, or limited resources (57, 58). High system costs can divert funds from essential care (59). Informed consent is difficult to obtain where literacy is low, and patients often cannot opt out without losing access to services (60). Power imbalances between international researchers and local communities, along with secondary data use, further threaten autonomy (61).

Exploitation risks include “data colonialism,” where LMICs supply data but gain little benefit, while intellectual property and profits accumulate to foreign institutions (62). AI systems may also overlook cultural beliefs and traditional healing practices, amplify disease-related stigma, or harm marginalized groups if community perspectives are ignored (63). Ethical AI deployment requires strong governance, equitable partnerships, robust infrastructure, community engagement, and local validation. LMICs should strengthen data protection laws, build regulatory and ethics review capacity, and establish clear accountability mechanisms. AI models trained on data from one region or population may not generalize well to another without careful customization and adaptation. This could be mitigated by using transfer learning models and explainable AI (64–66). Finally, AI tools must be rigorously tested in local settings, continuously monitored for bias, and adapted based on real-world performance and feedback.

## 4 Artificial intelligence-driven tools to strengthen pandemic preparedness

The literature identifies several systematic barriers to One Health integration in pandemic preparedness systems across LMICs (Table 2) (67–70). AI-enabled tools may provide scalable solutions to bridge these implementation deficits.

**Table 2:** Key limitations of one health integration for pandemic preparedness in LMICs and its perceived priority.

Limitation category	Specific barriers	Impact on implementation	Priority
Resource and financing	Limited budgets for multi-sector approaches	One Health seen as lower priority than urgent health needs	High
	Funding constraints for integrated systems	Difficulty justifying cross-sector investments	High
	Competing development priorities	Resources diverted to immediate needs	High
Governance and political	Weak political will and leadership	Lack of high-level commitment to One Health	High
	Absent regulatory frameworks	No legal basis for cross-sector coordination	High
	Poor consensus on priority setting	Different sectors pursue conflicting agendas	Medium
Sectoral collaboration	Limited cross-sector integration	Veterinary and environmental sectors poorly integrated	High
	Conflicting mandates and priorities	Different ministries have incompatible goals	High
	Poor data sharing and coordination	Information silos prevent integrated analysis	High
Capacity and infrastructure	Insufficient skilled One Health personnel	Lack of trained professionals for integrated approaches	High
	Weak laboratory systems	Limited diagnostic capabilities across sectors	High
	Poor data infrastructure	Inadequate systems for multi-sector data integration	Medium
Knowledge and awareness	Limited understanding of One Health concepts	Stakeholders do not grasp interconnected health benefits	Medium
	Lack of training programs	No systematic capacity building for One Health	Medium
	Professional silos	Disciplines work in isolation without collaboration	Medium
Health system fragmentation	Disjointed surveillance systems	Separate monitoring for human, animal, environment	High
	Institutional silos	Organizations operate with segmented mandates	High
	Overlapping or gap-ridden responsibilities	Unclear roles create inefficiencies	Medium
Geographic and access	Limited rural area coverage	Remote regions lack One Health services	High
	Poor connectivity and infrastructure	Inadequate technology for integrated monitoring	Medium
	Difficult terrain and logistics	Physical barriers to comprehensive coverage	Medium
Priority balancing	Competing urgent healthneeds	One Health competes with immediate health crises	Medium
	Economic development pressures	Activities causing disease risk contribute to GDP	High
	Short-term vs. long-term planning	Immediate needs overshadow prevention investments	Medium

### 4.1 Resource and financing optimization

The development of cost-effective solutions can be facilitated by AI-powered resource allocation models that can optimize limited budgets by identifying highest-impact interventions, demonstrate return on investment of One Health investments through predictive modeling, and eliminate costs of physical infrastructure for data collection and sharing. Machine learning can further optimize labor by using supervised learning and reinforcement learning techniques (71). AI-frameworks can generate real-time compelling evidence of cost-effectiveness: using AI-driven smart devices can provide simple and low-cost alternatives to standard RT-PCR detection, which is costly, time-consuming, and requires specific materials and equipment (72).

### 4.2 Governance and political barriers

Governance and political barriers are often intertwined with economics. When looking at One Health-related economics, a key element is the need of multi-sectoral economic impact assessment to identify extended relevance and possible resource-sharing (73). AI dashboards provide real-time evidence to policymakers showing cross-sector health threats, enabling informed decision-making based on current conditions rather than outdated reports (74). Predictive models can demonstrate potential economic losses from inaction, creating compelling evidence that motivates political will and justifies investment in One Health approaches (75). Automated data analysis and reporting systems ensure consistent documentation for international compliance, reducing the administrative burden on resource-constrained governments (76). Scenario modeling capabilities help policymakers understand the consequences of different policy choices, allowing them to evaluate trade-offs and optimize interventions before implementation. Beyond policy support, AI facilitates coordination across traditionally silo government sectors through platforms that automatically integrate data from different ministries, breaking down information barriers that often impede collaborative governance (77). Furthermore, federated learning offers a viable approach for governments to share data insights while maintaining privacy and sovereignty, enabling them to train models locally and exchange only the model parameters rather than raw data (78). Natural language processing can translate between different sector terminologies and priorities, enabling veterinary, health, and environmental professionals to communicate more effectively despite their specialized vocabularies (79).

### 4.3 Sectoral collaboration improvement

AI can break down data silos between human health, veterinary, and environmental sectors by automatically integrating and standardizing information across these traditionally separate systems (80). Machine learning (ML), has been used in the public sector to enhance collaboration, albeit with challenges around data sharing, privacy and security concerns that need to be overcome (81). Within One Health, ML algorithms can identify critical patterns requiring cross-sector response before humans recognize these connections, providing early warning capabilities that prevent localized threats from escalating (82). Automated alert systems ensure rapid coordination when thresholds are met, while digital workflows standardize collaboration processes across ministries, reducing confusion from incompatible procedures. Tools like chat bots, machine learning-driven task ticketing systems, robotic process automation, and natural language processing NLP have been described for IT systems (83).

### 4.4 Capacity and infrastructure gaps

AI-powered diagnostic tools can extend capabilities of limited trained personnel. Although limitations for optimal, safe, and equitable use of Chat bots exist, they could provide additional 24/7 guidance to field workers in remote areas with basic expertise (84). Automated training modules can scale capacity building without requiring expert trainers. For laboratory enhancement, tools for automated analysis of histopathology slides and automation of routine diagnostic tasks can ease pathogen identification in under-resourced labs (85, 86). Cloud-based AI provides sophisticated analysis capabilities without expensive local equipment. For instance, COVID-19 CT or tuberculosis X-rays screening tools (50). Predictive maintenance analytics can optimize equipment uptime in resource-constrained settings by predicting impending equipment usage, failures, and correlating multiple sensor inputs for comprehensive analysis (87). The underlying assumption for the optimal use of these tools is, however, that a reliable internet connectivity is available.

### 4.5 Knowledge and awareness building

AI-powered education and training systems address knowledge gaps in One Health implementation through personalized learning platforms that adapt content to different professional backgrounds. Multilingual AI assistants can bridge language barriers, and virtual reality simulation platforms can train health staff for safe emergency response practice (88–91). These systems democratize access to One Health knowledge by overcoming linguistic barriers and resource constraints while capturing institutional learning for wider dissemination through extension services to rural folk and general mass. Complementary AI-enhanced stakeholder engagement strategies expand One Health awareness through tailored visualizations for diverse audiences, social media analytics to identify influential voices and optimize messaging (92) besides involving local clubs and mass organizations. Telemedicine connects patients with human healthcare providers remotely through video calls, messaging, and monitoring devices (93). Telemedicine and AI tools serve different but complementary functions in healthcare. AI tools are software systems that automatically analyze data, recognize patterns, and provide recommendations without requiring real-time human interaction—operating 24/7 to process large datasets (94). The key difference lies in human involvement: telemedicine facilitates remote human-to-human medical care, while AI augments human capabilities through automated analysis.

### 4.6 Health system integration

The One Health sectors produce a vast variety of data formats that, merged, could significantly ease pandemic preparedness frameworks. AI is already in use for some of them. For instance the IQ Air system leverages AI to process data from a global network of over 25,000 air quality sensors spanning more than 140 countries, delivering immediate insights into air quality impacts (95). The xView2 program combines machine learning and computer vision with satellite imagery to identify buildings damaged in natural disasters, reducing danger and saving time for first responders who would otherwise make manual assessments, while helping search-and-rescue teams quickly identify priority areas (96). Health Map, an internet-based infectious disease surveillance system, has identified emerging infectious disease outbreaks including influenza A (H1N1) for over a decade, using continuously expanding dictionaries in more than nine languages to extract location-based data and detect disease outbreak patterns in real-time (97).

AI can also address health system fragmentation through cost-effective middleware that connects disparate systems without expensive overhauls. Data standardization and harmonization could be performed using Python libraries with declarative, unified APIs for standardizing different column types, simplifying the LLM’s code generation with concise API calls (98). Precision medicine frameworks can help clinical workflow automation and eliminates gaps and overlaps in information flow (98–100). Federated learning enables cross-sector collaboration while maintaining data sovereignty by not needing raw data sharing, particularly important for international cooperation. AI-powered performance monitoring complements these solutions by identifying systemic inefficiencies, predicting potential system failures for proactive intervention, and providing real-time integrated dashboards that give decision-makers comprehensive visibility across human, animal, and environmental health domains. This can ease a coordinated response and evidence-based resource allocation.

### 4.7 Accessibility solutions

**Figure 1:**
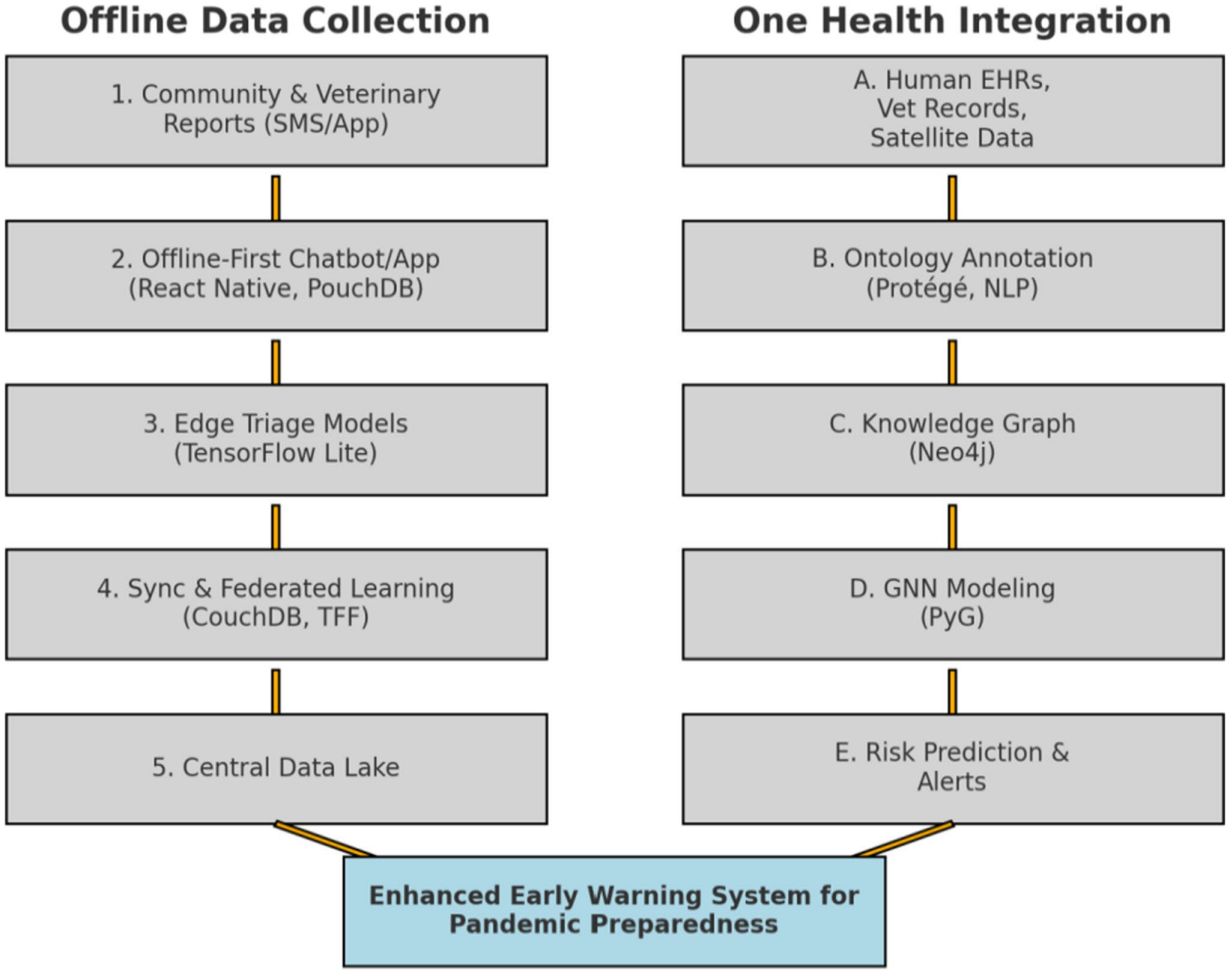
Example of a dual solution combining offline data collection and one health integration into an early warning system for pandemic preparedness.

AI technologies overcome geographic barriers in One Health implementation by providing comprehensive remote area support where zoonotic disease risks are highest but traditional surveillance is weakest. Satellite imagery analysis continuously monitors environmental health risks and spillover indicators like deforestation and wildlife shifts (101, 102). Drone-based surveillance extends monitoring to inaccessible regions for wildlife health and environmental assessment (103), and telemedicine platforms could connect remote workers with specialists for expert guidance on complex zoonotic cases (103, 104). In agriculture, several AI-driven tools for disease forecasting exists (105). AI weather and climate models can predict disease-favorable conditions. IoT sensors with AI analysis could help with mobile-based syndromic surveillance, addressing the critical gap where high-risk human-animal-environment interfaces occur in geographically isolated regions across LMICs. AI can help bridge these gaps by serving as a force multiplier that extends limited human and financial resources, automates routine tasks, and provides sophisticated analytical capabilities that would otherwise be unaffordable for many LMICs. The key is implementing AI solutions that are appropriate to local contexts and sustainable within existing resource constraints. A dual solution to the gaps could be proposed to exemplify this (Figure 1). The first component can be a Graph Neural Network (GNN)-based model that can integrate human health records, veterinary reports, and environmental data such as deforestation or climate anomalies into a unified AI-based risk assessment system. By representing these elements as nodes and relationships in a heterogeneous knowledge graph, the GNN could help predict where and when diseases and spillover events are likely to occur (26, 106). The second component can represent a simple, offline-first digital chat bot and mobile app designed specifically for LMIC use. Mobile Health platforms have been previously described as affordable and scalable, making them particularly valuable for LMICs facing budgetary limitations (107–109). This approach can enable health workers, farmers, and community members to report symptoms and animal deaths without requiring constant internet access. These reports are stored locally, synced when basic connectivity is available, and fed into the central GNN model to ensure near real-time updates of national risk maps.

## 5 Discussion and future directions

Global pandemic preparedness continues to face challenges due to fragmented surveillance systems that fail to integrate human, animal, and environmental health data—a critical gap highlighted by WHO. AI-driven technologies can help implement a One Health approach to pandemic preparedness in resource-limited settings. However, LMICs are particularly affected due to infrastructural, cultural, and capacity-based limitations. One Health-integrated frameworks need to be tailored to the specific local settings (29, 110).

Foundation-level priorities for One Health implementation should focus first on building political will and governance frameworks, as without political commitment, all other interventions will fail. Immediate actions include securing high-level government endorsement and establishing legal and regulatory frameworks (37). Equally critical issues are resource and financing mechanisms that provide the foundation for sustainable implementation through establishing dedicated One Health budget lines and securing international funding commitments (68). Most importantly, AI tools should be audited systematically, to ensure they operate fairly, accurately, and transparently. The building of regulatory capacity for AI oversight is therefore critical. Following these elements, basic infrastructure and systems integration becomes essential as it provides the technical foundation for surveillance and response capabilities, enabling operational implementation. Finally, expansion of geographic coverage and access should be pursued, as it requires established systems to scale effectively.

The successful and ethical deployment of AI for infectious disease management in LMICs requires moving beyond a purely technical approach to address the complex social, economic, and political contexts in which these systems operate. Solutions must be co-designed with local stakeholders, respect community values, and strengthen rather than undermine existing healthcare systems (111). Engaging local communities, ensuring transparency, and providing user training are, therefore, essential prerequisites for system acceptance and success.

Future research should prioritize pilot testing of this integrated system in partnership with local stakeholders, co-designing solutions that are culturally appropriate and socially trusted. Additionally, the implementation strategy should be phased. Mobile/cloud-based solutions requiring minimal local infrastructure should be prioritized. A focus should be put on high-impact, low-cost applications like syndromic surveillance (112, 113). The building of capacity should, whenever possible, utilize existing systems rather than creating entirely new infrastructure. Together, the described approaches offer a promising step toward a unified, real-time, and equitable surveillance framework. However, successful implementation will depend not only on technical performance but also on cultural acceptance, local capacity building, and ethical deployment, ensuring that even resource-limited regions become active participants in global pandemic resilience. To achieve this, priority should be given to address the political will to invest in AI-tools, professional training of local implementers in the public health sector, and the expansion of local infrastructure. With these elements in place, the AI-driven One Health integration into pandemic preparedness will be actionable innovation.
